# High-throughput sequencing of small RNAs and anatomical characteristics associated with leaf development in celery

**DOI:** 10.1038/srep11093

**Published:** 2015-06-09

**Authors:** Xiao-Ling Jia, Meng-Yao Li, Qian Jiang, Zhi-Sheng Xu, Feng Wang, Ai-Sheng Xiong

**Affiliations:** 1 State Key Laboratory of Crop Genetics and Germplasm Enhancement, College of Horticulture, Nanjing Agricultural University, Nanjing, 210095, China

## Abstract

MicroRNAs (miRNAs) exhibit diverse and important roles in plant growth, development, and stress responses and regulate gene expression at the post-transcriptional level. Knowledge about the diversity of miRNAs and their roles in leaf development in celery remains unknown. To elucidate the roles of miRNAs in celery leaf development, we identified leaf development-related miRNAs through high-throughput sequencing. Small RNA libraries were constructed using leaves from three stages (10, 20, and 30 cm) of celery cv.‘Ventura’ and then subjected to high-throughput sequencing and bioinformatics analysis. At Stage 1, Stage 2, and Stage 3 of ‘Ventura’, a total of 333, 329, and 344 conserved miRNAs (belonging to 35, 35, and 32 families, respectively) were identified. A total of 131 miRNAs were identified as novel in ‘Ventura’. Potential miRNA target genes were predicted and annotated using the eggNOG, GO, and KEGG databases to explore gene functions. The abundance of five conserved miRNAs and their corresponding potential target genes were validated. Expression profiles of novel potential miRNAs were also detected. Anatomical characteristics of the leaf blades and petioles at three leaf stages were further analyzed. This study contributes to our understanding on the functions and molecular regulatory mechanisms of miRNAs in celery leaf development.

Celery (*Apium graveolens* L.) belongs to the Apiaceae family and biennial herbs. Although celery originated from the Mediterranean, it is now cultivated and consumed worldwide^1^. Celery is rich in carotenoids, flavonoids, volatile oils, vitamins, folic acid, and dietary fiber^2^,^3^. Celery was initially grown for medicinal use because of its beneficial effects on the digestive and cardiovascular systems^4^. ‘Ventura’ originated from the United States and was later introduced to China. This cultivar compacts with thick glossy leaves and is well known for its high disease resistance and yield.

In vascular plants, leaves serve important functions in growth and biomass production through photosynthesis and transpiration during development. The leaves (petioles and leaf blades) are the main edible parts in celery. Many complex genetic signals and interactions are involved in cell fate during leaf development. Numerous studies indicated that leaf development is regulated by microRNAs (miRNAs)^5^,^6^,^7^. The overexpression of miR396 can decrease growth-regulating factors (GRFs), which affect cell proliferation in the meristem and developing leaves of *Arabidopsis thaliana*^8^. The regulation of *lanceolate* (*LA*) by miR319 is required for compound-leaf development in tomatoes^6^. Gradual increase of miR156 regulates temporal expression changes of numerous genes during leaf development in rice^5^.

miRNAs are an abundant new class of non-coding endogenous ~20 nt to 24 nt small RNAs^9^ that widely exist in animals^10^, plants^11^, and some viruses^12^. This new class of small RNAs has diverse and important roles in plant development, protein degradation, stress responses, signal transduction, and metabolism^13^,^14^. The mature miRNAs are located in hairpin structures and are generated from primary miRNAs (pri-miRNAs) via at least two RNAseIII-mediated steps^15^. miRNAs do not directly regulate plant growth and development, but regulate gene expression at the post-transcriptional level^9^,^16^.

miRNAs play important roles in leaf development in higher plants. Leaf polarity is regulated by the miR390/ARF pathway^17^, stomatal patterning is regulated by miR824 via *Agamous-like16* (*AGL16*)^18^, and leaf shape is regulated by miR396 via *GRF*^19^. The *CINCINNATA* (*CIN*) gene regulated by miR319/Jaw is required for the differential regulation of cell division during leaf morphogenesis to obtain flat leaves^20^,^21^. miR165 and miR166 are essential for controlling leaf patterns (radialization and adaxialization) and for vascular bundle development of leaves by directly regulating homeodomain–leucine zipper (HD-ZIP) transcription factor genes^22^,^23^,^24^.

We previously reported 469 (457 known and 12 novel), 431(418 known and 13 novel), and 346 (341 known and 5 novel) miRNAs of celery varieties, namely, ‘Liuhe Huangxinqin’, ‘Jinnan Shiqin’, and ‘Ventura’ by deep sequencing, respectively^25^,^26^. However, knowledge on miRNAs at developmental stages of celery leaves remains unknown. In this study, small RNA libraries were constructed using celery leaves from the three stages and were subjected to deep sequencing and bioinformatics analysis. Five conserved miRNAs associated with celery leaf development and their corresponding potential target genes were identified, meanwhile five novel potential miRNAs with high count were also selected. Their expression profiles were determined at different stages of leaf development by using quantitative real-time PCR (qRT-PCR). The petioles and leaf blades at three stages were also anatomically characterized. Results provide valuable information to elucidate the miRNAs associated with leaf development in celery.

## Results

### Anatomical characteristics of leaf blades and petioles from ‘Ventura’ at three stages

**Figure 1:**
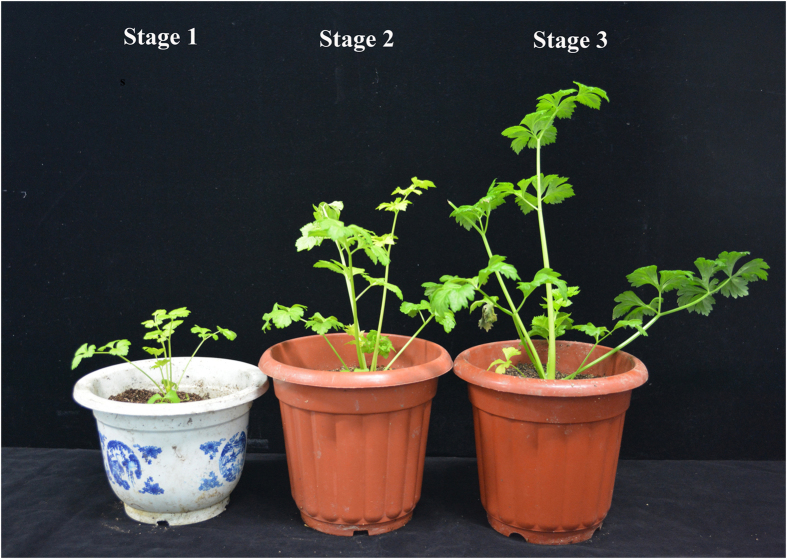
‘Ventura’ at the three stages. The length of the leaves at Stage 1 was 10 cm (35 d), the length of the leaves at Stage 2 was 20 cm (50 d), and the length of the leaves at Stage 3 was 30 cm (65 d).

**Figure 2:**
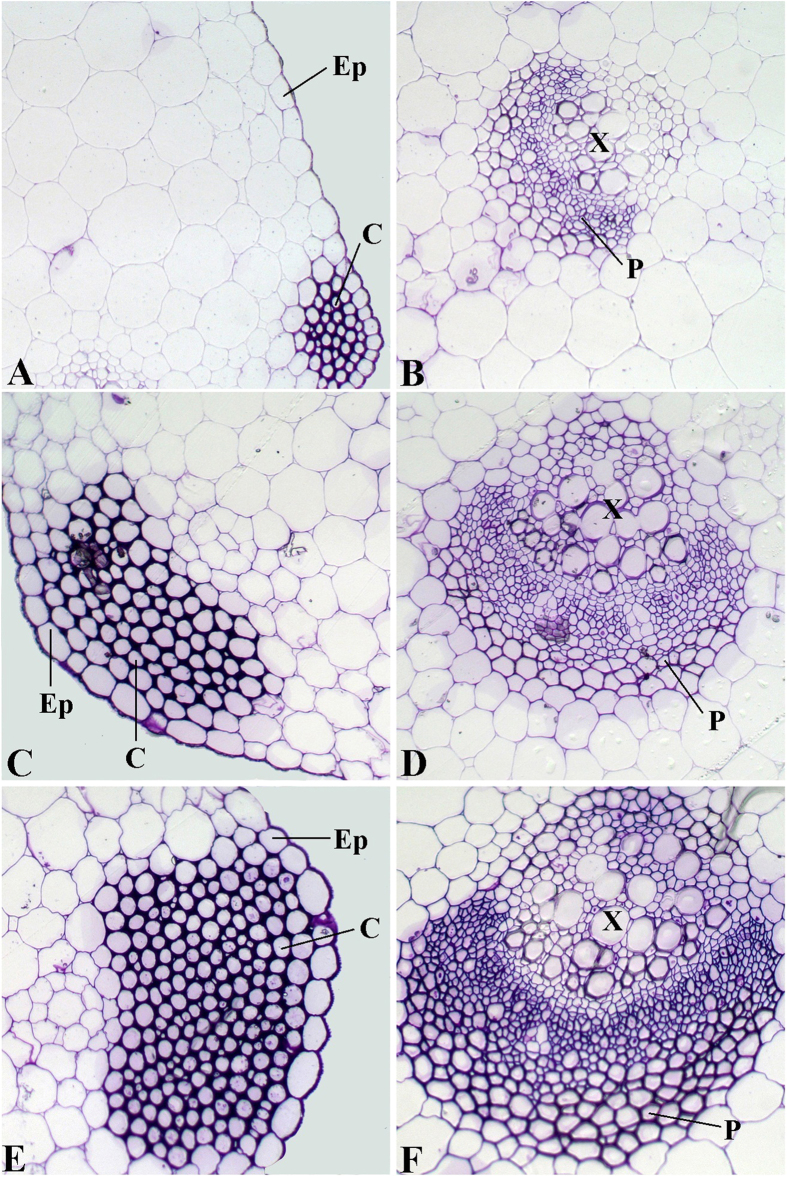
Structural comparison of ‘Ventura’ petioles. The petiole specimens were cut from the middle part. The specimens were stained with 0.5% methylviolet. A, B: Stage 1 of ‘Ventura’ ×200; C, D: Stage 2 of ‘Ventura’ ×200; E, F: Stage 3 of ‘Ventura’ ×200. Ep: epidermis; C: collenchyma; P: phloem; X: xylem.

**Figure 3:**
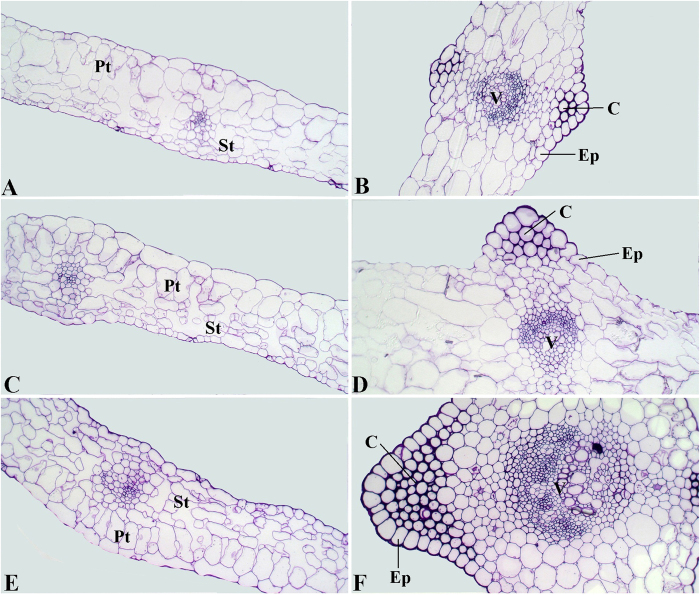
Structural comparison of ‘Ventura’ leaf blades. The leaf blade specimens were cut from the main leaf vein and mesophyll tissue near the vein. The specimens were stained with 0.5% methylviolet. A: Mesophyll at Stage 1 × 200; B: Leaf vein at Stage 1 × 200; C: Mesophyll at Stage 2 × 200; D: Leaf vein at Stage 2 × 200; E: Mesophyll at Stage 3 × 200; F: Leaf vein at Stage 3 × 200. Pt: palisade tissues; St: spongy tissues; Ep: epidermis; V: vascular bundles; C: collenchyma.

The leaf structure at three development stages (Fig. 1) in ‘Ventura’ was comprehensively investigated using resin-embedded microtome. As shown in Fig. 2, the collenchyma grew stronger and epidermal cells gradually expanded from Stage 1 to Stage 3. The phloem and xylem extensively developed, and vascular bundles expanded at the three stages. As shown in Fig. 3, the spongy and palisade mesophyll tissues were gradually and tightly arranged and further developed from Stage 1 to Stage 3. The collenchyma and vascular bundles in the leaf vein further expanded during leaf growth and development.

### High-throughput sequencing analysis of small RNAs extracted from celery leaves

**Figure 4:**
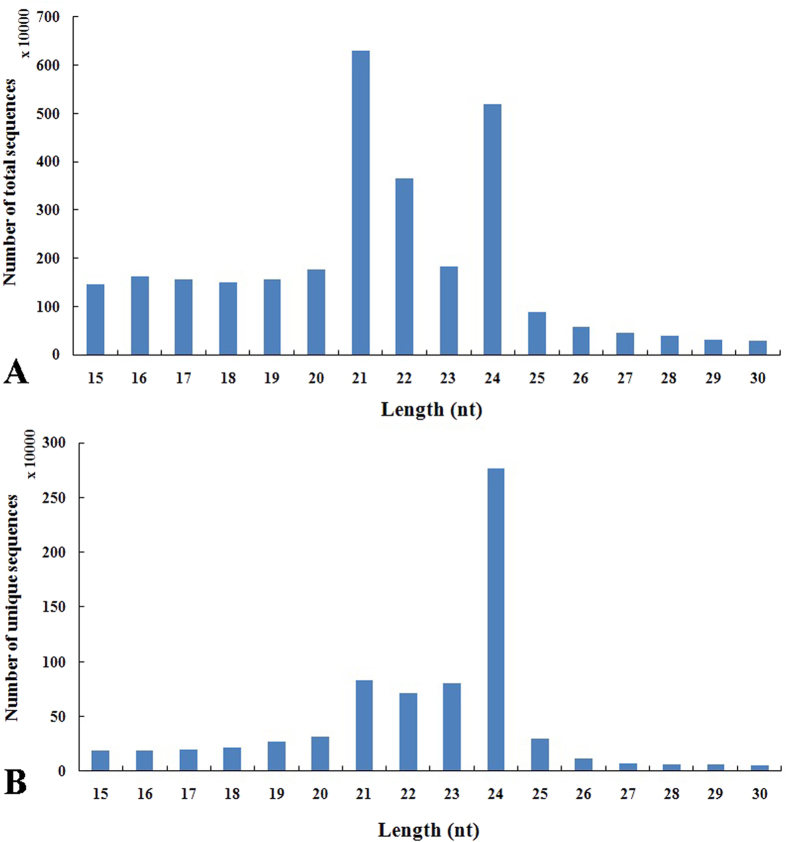
Length distribution of small RNAs in ‘Ventura’. The reads with a base quality less than 20 were removed, and sequences ≤15 nt in length were discarded. Sequences with 15 nt to 30 nt were used for analyses. A: Size distribution of the total small RNA sequences; B: Size distribution of the unique small RNA sequences.

To identify miRNAs involved in the development of celery leaves, we performed high-throughput sequencing of small RNA libraries constructed from the three leaf development stages, as follows: Stage 1 (35 d), Stage 2 (50 d), and Stage 3 (65 d). After filtering the low-quality reads, 3’ insert null, poly (A), and reads with <15 or >30 length, we obtained 7,400,022 clean reads at Stage 1, 8,157,893 clean reads at Stage 2, and 13,777,047 clean reads at Stage 3. A total of 29,334,962 clean reads and 7,156,946 unique sequences were obtained. The length of small RNAs varied from 15 nt to 30 nt, and the majority of the reads were 21 nt to 24 nt in length (Fig. 4). In the size distribution of the total reads, the 21 nt-long sequence size was the most abundant, followed by 24 nt. Analysis of the size distribution of the unique sequences revealed that the 24-nt small RNAs were the most abundant, followed by 21 nt. This result is consistent with previous reports on other species, such as *A. thaliana*, cucumber, tomato, trifoliate orange, maize, and peanut^27^,^28^,^29^,^30^,^31^. Previous studies also reported that 21 nt siRNAs characterize authentic miRNAs, and 24 nt siRNAs are associated with repeats and heterochromatic modifications^32^.

**Figure 5:**
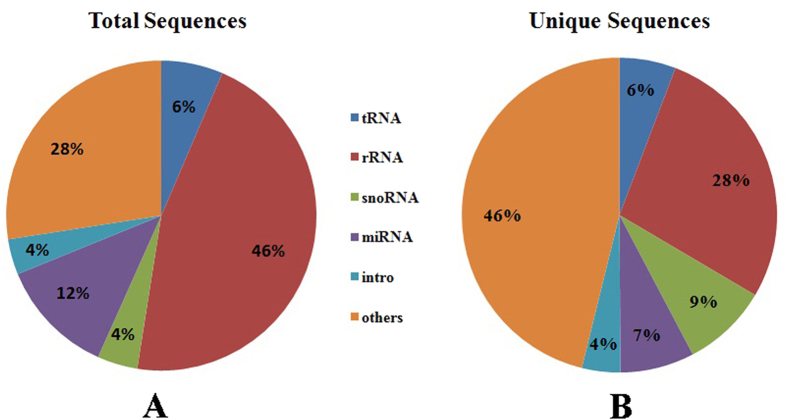
Distribution of small RNAs among different categories in ‘Ventura’. The clean reads were annotated as tRNAs, rRNAs, snoRNAs, miRNAs, intro, and others based on the Rfam database. A: Distribution of the total small RNA sequences among different categories in ‘Ventura’; B: Distribution of the unique small RNA sequences among different categories in ‘Ventura’.

**Figure 6:**
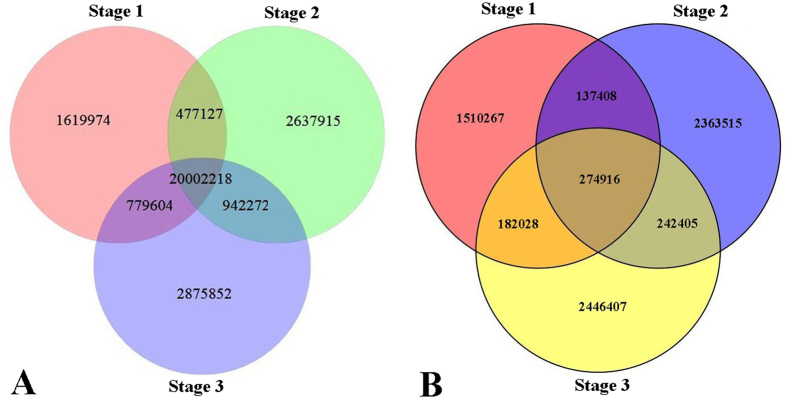
Venn diagrams showing the number of total and unique sequences at the three stages. A: Number of the total sequences at the three stages; **B**: Number of the unique sequences at the three stages.

The different types of RNA sequences were further classified by performing BLAST searches against the Rfam database. These sequences included miRNA, rRNA, tRNA, snoRNA, intro, and other unannotated reads (Fig. 5). Generally, the distribution of the total small RNA sequences and unique small RNA sequences slightly differed in ‘Ventura’. The distribution of the total and unique sequences at the three stages is shown in Fig. 6.

### Identification of conserved and novel miRNAs in celery

**Figure 7:**
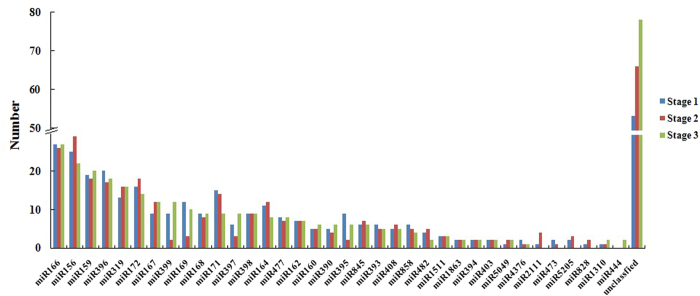
Numbers of identical miRNA members in each family in ‘Ventura’. Small RNA sequences were compared with currently known miRNAs in the miRNA database miRbase.

**Figure 8:**
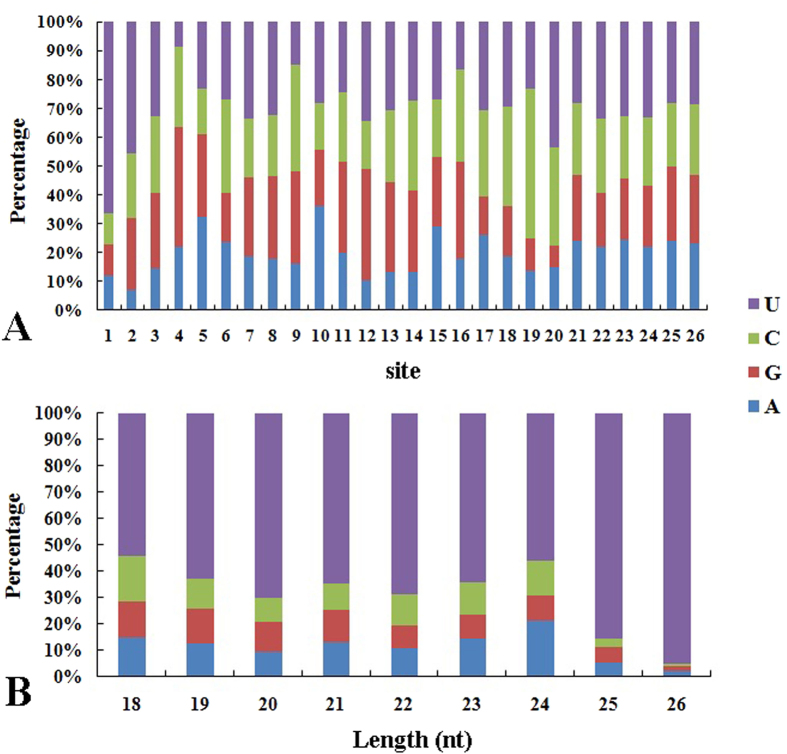
Base bias of miRNAs in ‘Ventura’. **A**: Base bias on the specific site of miRNAs in ‘Ventura’; **B**: Base bias on the first site of miRNAs with specific lengths in ‘Ventura’.

To identify conserved miRNAs in ‘Ventura’, we compared all small RNA sequences with currently known miRNAs in the miRNA database miRbase. A total of 333, 329, and 344 unique miRNA sequences (belonging to 35, 35, and 32 miRNA families) were identified in ‘Ventura’ at Stage 1, Stage 2, and Stage 3, respectively (Fig. 7). The base bias on a specific site of miRNAs and the first site of miRNAs with specific lengths are shown in Fig. 8. The majority of these miRNAs started with a 5’-U, which is consistent with typical miRNA sequence patterns^33^.

To identify novel miRNA sequences, we mapped all unannotated small RNAs onto the celery transcriptome sequence data (SRX326597). Potential pre-miRNAs were searched, and hairpin-like structures were predicted. A total of 131 unique sequences were identified as novel miRNAs in ‘Ventura’ (Supplementary File 1). The average minimum free energy value in ‘Ventura’ was −23.63 kcal/mol, which indicated high stability in the hairpin structures.

### Prediction and annotation of miRNA putative target genes

**Figure 9:**
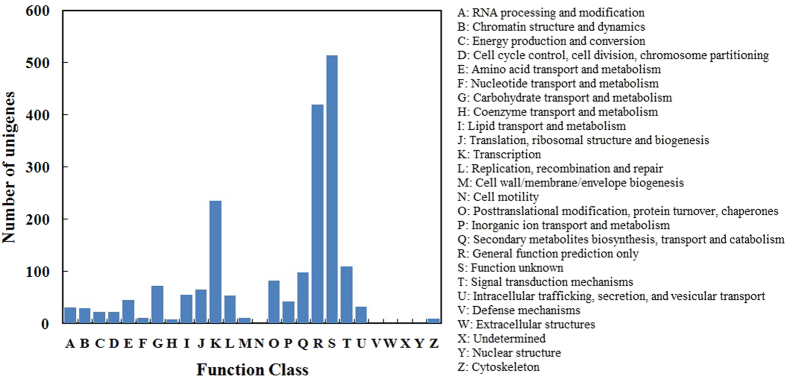
eggNOG classification assigned to miRNA targets in ‘Ventura’. miRNA target genes were assigned based on eggNOG database.

**Figure 10:**
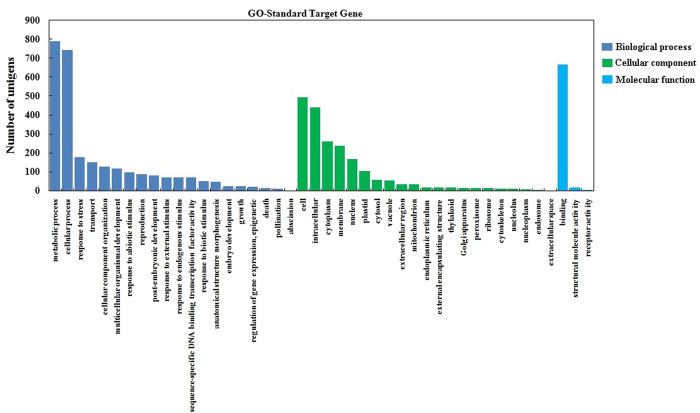
GO classification of miRNA targets in ‘Ventura’. miRNA target genes were assigned by using Blast2GO.

**Figure 11:**
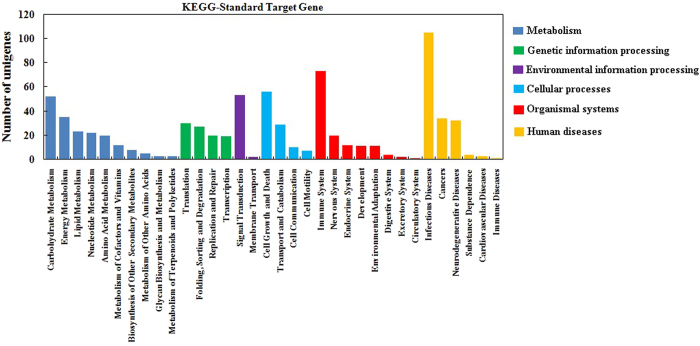
KEGG classification of miRNA targets in ‘Ventura’. miRNA target genes were assigned based on the KEGG database by using BLASTx.

miRNA putative target genes were predicted using the psRNA target program, and 1,432 potential target genes were identified in ‘Ventura’. To evaluate the completeness of the transcriptome library and the effectiveness of the annotation process, we searched the miRNA target genes against the eggNOG database for functional prediction and classification (Fig. 9)^34^. A total of 1,974 sequences were assigned to eggNOG classification, and they were functionally classified into 26 eggNOG categories. The cluster for ‘function unknown’ was the largest group, followed by ‘general function prediction only’ and ‘transcription’. To describe the miRNA target gene products in terms of their associated biological processes, cellular components, and molecular functions, we conducted GO analysis by using Blast2GO (Fig. 10)^35^. A total of 2,019 target genes were categorized as cellular component, 2,951 as biological process, and 800 as molecular function. ‘Metabolic process’ and ‘cellular process’ were the most highly represented groups under the biological process category. For the cellular component category, ‘cell’ and ‘intracellular’ were the most highly represented groups. With regard to molecular function, ‘binding’ was the most highly represented group. The miRNA target genes were assigned based on the KEGG database to special biochemical pathways by using BLASTx (Fig. 11)^36^. Thirty-four different pathways were found, and the most frequently represented pathway was ‘infectious diseases’, followed by ‘immune system’.

### Expression profiles of conserved miRNAs and their potential target genes in the petioles and leaf blades at different stages of ‘Ventura’

**Figure 12:**
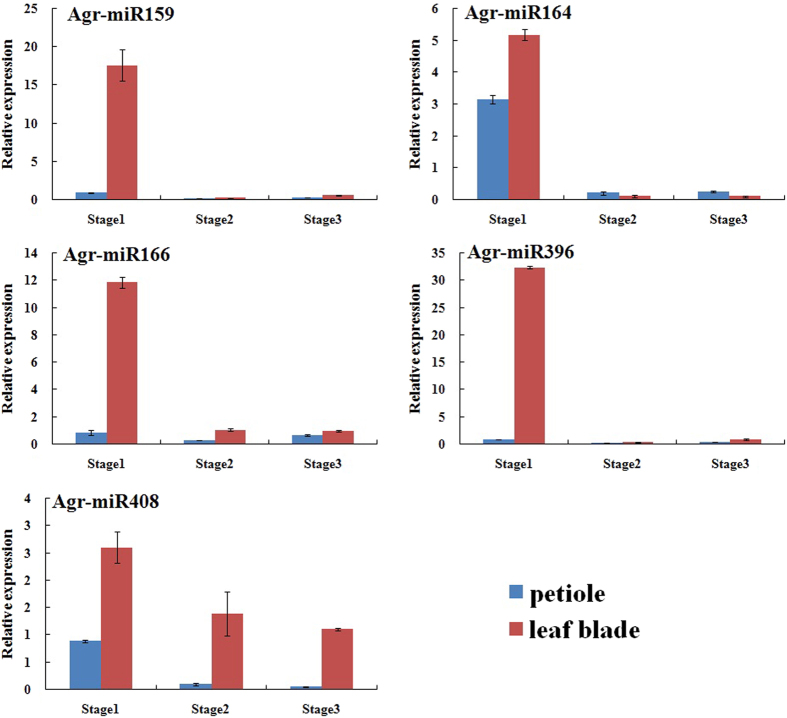
Expression profiles of conserved miRNAs in the petioles and leaf blades at different stages of ‘Ventura’. Each bar represents the mean value from triplicate experiments ±SD.

**Figure 13:**
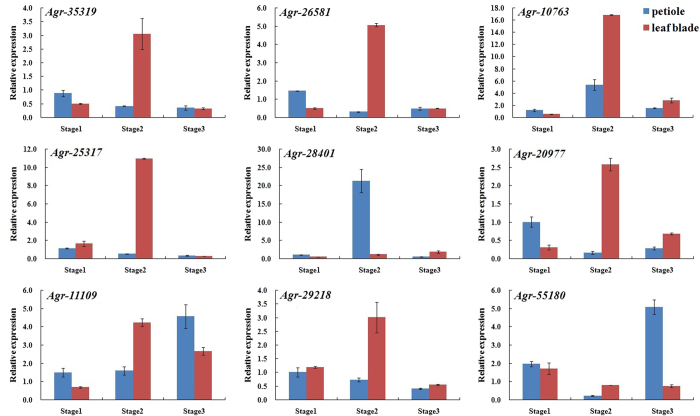
Expression profiles of potential target genes in the petioles and leaf blades at different stages of ‘Ventura’. Each bar represents the mean value from triplicate experiments ±SD.

To investigate the regulatory function and mechanism of the conserved miRNAs and their potential target genes during celery leaf development, the expression of five conserved miRNAs and their potential target genes were validated by qRT-PCR in the petioles and leaf blades at different stages of ‘Ventura’ (Figs 12 & 13). High-throughput sequencing could estimate the expression profiles of miRNA genes. We selected five highly expressed conserved miRNAs from each miRNA family according the high-throughput sequencing data, namely Agr-miR159, Agr-miR164, Agr-miR166, Agr-miR396, and Agr-miR408.

In this study, five conserved miRNAs related to leaf development were expressed in the petioles and leaf blades of ‘Ventura’ at the three stages (Fig. 12). In the petioles, the relative transcript level of Agr-miR408 increased and peaked at Stage 1, decreased at Stage 2, and then continued to decrease and diminish at Stage 3. The relative expression levels of Agr-miR159, Agr-miR164, Agr-miR166, and Agr-miR396 decreased at Stage 2, but increased at Stage 3. The transcript levels were lower at Stage 3 than at Stage 1. In the leaf blades, the transcript levels of Agr-miR159, Agr-miR164, Agr-miR166, Agr-miR396, and Agr-miR408 gradually decreased at the three stages. The transcript levels of Agr-miR159, Agr-miR164, Agr-miR166, and Agr-miR396 at Stage 2 and Stage 3 were low, and no significant difference was found at these two latter stages. These results indicated that these five miRNAs are regulated during celery leaf development.

Those five selected miRNAs (Agr-miR159, Agr-miR164, Agr-miR166, Agr-miR396, and Agr-miR408) targeted their corresponding potential genes (*Agr-35319* and *Agr-26581* gene, *Agr-10763* and *Agr-25317* gene, *Agr-28401* and *Agr-20977* gene, *Agr-11109* and *Agr-29218* gene, *Agr-55180* gene), respectively. There is no obvious regularity of the expression levels of their corresponding potential target genes during the 3 stages. By comparing the expression profiles of the five conserved miRNAs and their corresponding potential target genes, we found that the expression of the target genes were independent with miRNAs (Fig. 13).

### Expression profiles of novel potential miRNAs in the petioles and leaf blades at different stages of ‘Ventura’

**Figure 14:**
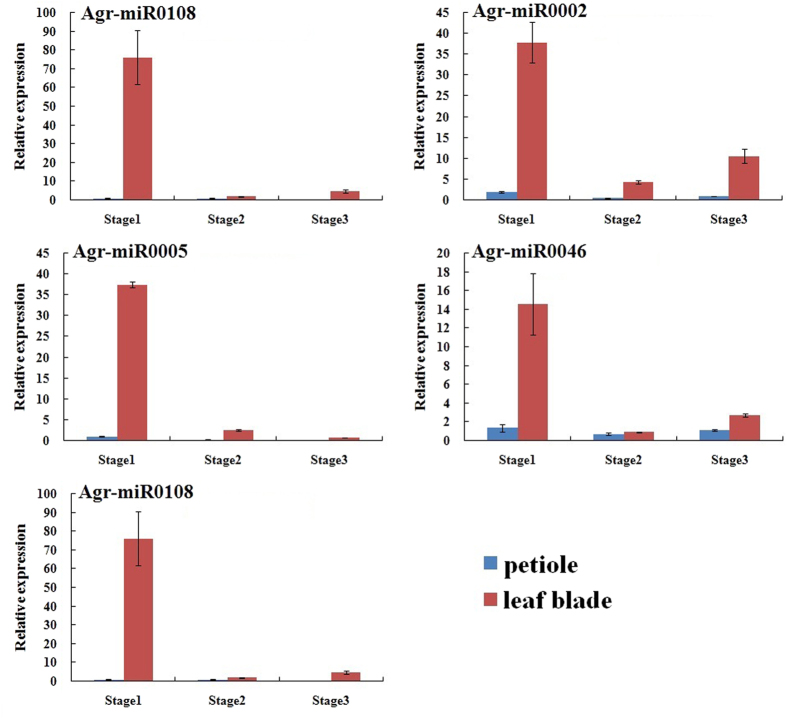
Expression profiles of novel potential miRNAs in the petioles and leaf blades at different stages of ‘Ventura’. Each bar represents the mean value from triplicate experiments ±SD.

In this study, the expression profiles of five novel potential miRNAs with high count (Agr-miR0056, Agr-miR0002, Agr-miR0005, Agr-miR0046 and Agr-miR0108) were also detected by qRT-PCR (Fig. 14). Significant differences in relative expression levels were measured in the petioles and leaf blades of celery at the 3 stages. The relative expression levels of the five novel potential miRNAs were much higher in the leaf blades than in the petioles of celery. The relative expression levels in the petioles were relatively low, and no significant difference was detected among the 3 stages of celery. The relative transcript level in leaf blades was the highest at Stage 1, and relatively low at Stage 2 and Stage 3.

## Discussion

miRNAs are important regulators of gene expression at the post-transcriptional level because they repress gene translation. miRNAs play vital roles in plant growth and development and under stressful conditions^37^,^38^,^39^. In this study, miRNAs were identified and characterized using leaves from the three stages of celery cv. ‘Ventura’ through high-throughput sequencing. A total of 333, 329, and 344 known miRNAs (belonging to 35, 35, and 32 families) were detected at Stage 1, Stage 2, and Stage 3, respectively. About 131 novel miRNAs were also identified in ‘Ventura’. The potential target prediction for miRNAs and detailed functional information are important aspects of this study. A total of 1,432 potential target genes were assigned to eggNOG^34^, GO^35^, and KEGG classifications^40^. These results provide useful information for further research on miRNAs that are related to leaf development in celery.

Numerous studies confirmed that small RNAs play important roles in leaf development in higher plants^5^,^41^. miRNAs negatively regulate meristem identity, cell division, organ separation, organ polarity, and other developmental processes^42^,^43^,^44^. In the present study, five known miRNAs (Agr-miR159, Agr-miR164, Agr-miR166, Agr-miR396, and Agr-miR408) associated with celery leaf development were identified using high-throughput sequencing of small RNAs. Leaf shape is regulated by miR396 via *GRF*^19^, and leaf pattern (radialization and adaxialization) is regulated by miR166 via HD-ZIP transcription factors^22^. miR164 is required to regulate the gene expression of *CUC 1* in developing leaf lamina^45^. miR159/JAW is critical for leaf development and cell division because it targets a subset of TCP transcription factor genes^20^,^46^. miR408 is predicted to target *LACCASE* gene, a regulator of lignin synthesis that is important in xylem and phloem development^47^,^48^. Moreover, miR166 targets HD-ZIP transcription factors and is required for leaf vascular bundle development^23^.

Gene regulation at different stages is closely related to celery leaf development^6^,^7^. qRT-PCR not only detects the existence of celery miRNAs, but also their expression trends at different stages. The five miRNA transcripts can be detected in the petioles and leaf blades at the three stages, with the highest expression levels observed at Stage 1. miRNAs also regulate gene expression at the post-transcriptional level by degrading or repressing the translation of targeted miRNAs^49^. GRF transcription factors mediate the effect on cell proliferation, leaf size, and longevity^7^. HD-ZIP transcription factors also regulate lateral organ polarity and meristem formation^50^. TCP transcription factor genes play important roles in cell division and signaling pathways that generate different leaf forms^51^. For example, the edge of leaves may be smooth or jagged, and these phenotypes are regulated by the CUC gene family^52^,^53^. As shown in Fig. 12, Agr-miR159, Agr-miR164, Agr-miR166, and Agr-miR396 transcripts in the petioles and leaf blades exhibited the highest expression levels at Stage 1. Therefore, we conclude that celery leaf development may be regulated by their target genes (GRF transcription factors, HD-ZIP transcription factors, TCP transcription factors, and the CUC gene family), with lower transcript levels at Stage 1 and higher transcript levels at the latter two stages.

Gene regulation at different stages is consistent with structural changes in plants. The transcript levels of Agr-miR408 in the petioles and leaf blades gradually decreased at the three stages. Agr-miR408 and Agr-miR166 transcripts showed the highest levels at Stage 1. *LACCASE*, a target of miR408, is required for lignin polymerization during vascular development in *Arabidopsis*^54^. HD-ZIP transcription factors, a target of miR166, also regulate vascular development^55^. ATHB15, a member of the HD-ZIP transcription factors, is mainly expressed in vascular tissues and plays important roles in plant vascular system development with expanded phloem and xylem tissues^23^. Consistent with the anatomical characteristics of the leaf blades and petioles at the three stages, the vascular bundles in the petioles and leaf vein further expanded and the phloem and xylem extensively developed.

Study of the target genes of miRNAs plays an important role in understanding the function and mechanism of miRNAs during leaf development of celery. miRNAs repressed the target gene expression primarily by translation inhibition and degradation of the mRNA. However, it was reported that translation regulation by microRNAs oscillated between repression and activation during the cell cycle^10^,^11^,^12^,^13^,^14^. Based on the results of the expression profiles of five conserved miRNAs and their potential target genes in the petioles and leaf blades at different stages of ‘Ventura’, the expression of the corresponding potential target genes was independent from miRNAs. Previous research also found that mutations of some genes compromise miRNA-mediated target gene repression at the protein level, instead of the mRNA. It can be concluded that the five conserved miRNAs regulate the corresponding potential target genes by translation inhibition.

The expression profiles of novel potential miRNAs in the petioles and leaf blades of celery at 3 stages might provide valuable information for the function of these miRNAs during celery leaf development. The data obtained showed potential evidence to support the existence of the novel potential miRNAs in celery. To better elucidate the function and mechanism of these novel potential miRNAs during celery leaf development, more studies need to be performed further.

miRNAs are a class of non-coding endogenous small RNAs that play diverse and important roles in plant growth, development, and stress responses. Many experiments demonstrated that miRNAs regulate leaf development, including leaf morphogenesis and polarity, and vascular development. miRNAs also regulate the expression of many important genes, and most of these genes are transcriptional factors that regulate plant development. In this research, we report a comprehensive study on celery miRNAs by using leaves from the three developmental stages through high-throughput sequencing and high-end bioinformatics methods. A population of small RNAs was characterized as conserved and novel miRNAs. The predicted target genes were subjected to eggNOG, GO, and KEGG annotation to infer their functions. The abundance of five conserved miRNAs and their corresponding potential target genes were validated by qRT-PCR in the petioles and leaf blades at different stages of ‘Ventura’. Expression profiles of novel potential miRNAs were also detected. The anatomical characteristics of the leaf blades and petioles at three leaf stages were analyzed by resin embedding with microtomy. Considering that the diversity of miRNAs and their roles in leaf development in celery remain ambiguous, this study provides useful information to elucidate the functions and molecular regulatory mechanisms of miRNAs in celery leaf development.

## Methods

### Plant materials

Celery cv. ‘Ventura’ seeds were deposited in the State Key Laboratory of Crop Genetics and Germplasm Enhancement, Nanjing Agricultural University, Nanjing, China. Celery plants were grown in pots containing peat and vermiculite (2:1,v/v) in a growth chamber with 16 h/8 h at 25 °C/15 °C for day/night, 3 000 lux of light intensity and 75% humidity. Fertilizer and water conditions were strictly controlled.

Three leaf development stages of celery were evaluated, and the heights of the celery at Stage 1, Stage 2, and Stage 3 were 10 cm (35 d), 20 cm (50 d), and 30 cm (65 d), respectively (Fig. 1). The leaves were collected at the three stages, immediately frozen in liquid nitrogen, and then stored at −80 °C until use. The petioles and leaf blades at the three stages were prepared for resin-embedded sections stained with methylviolet.

### Resin embedding with microtomy

Specimens were obtained from the petioles and leaf blades at the three stages by using a razor blade. The petiole specimens were cut from the middle part, and the leaf blade specimens were cut from the main leaf vein and mesophyll tissue near the vein. The specimens were prefixed with 2.5% glutaraldehyde at 4 °C overnight. The blocks were washed and dehydrated with an ethanol series of 30% to 100% and then embedded in Spurr low-viscosity embedding medium. Sections (1 μm thick) were cut with a glass knife in a Leica UltracutR microtome (Germany) and then stained with 0.5% methylviolet for 10 min. The sections were visualized and photographed using a CCD camera under Leica DMLS light microscope.

### RNA extraction, small RNA library construction, and sequencing

Small RNAs were extracted from the leaves at the three stages by using TRIZOL method to construct small RNA libraries. Three small RNA samples were sequenced with an Illumina Hi-seq 2000 platform (Shanghai Personal Biotechnology Co., Ltd.).

### Bioinformatics analysis of small RNA sequences

Sequencing reads were generated from the three constructed small RNA libraries. The raw sequences were subjected to bioinformatics analysis to process and identify the sequences representing conserved and novel miRNAs^56^. The low-quality sequences (reads with a base quality less than 20) were removed, and all sequences ≤15 nt in length were discarded. Sequences with 15 nt to 30 nt were used for further analyses. The clean reads were annotated as tRNAs, rRNAs, snoRNAs, miRNAs, intro, and other unannotated reads according to the Rfam (11.0 release) database. The unique sequences were used for BLASTN search against the miRNA database (miRBase16.0), and the perfectly matched sequences were identified as conserved miRNAs. Potential novel sequences were identified through alignment with celery transcriptome data.

### Prediction of potential miRNA target genes and functional annotation

The potential target genes of celery miRNAs were predicted using the psRNA target program (http://plantgrn.noble.org/psRNATarget/?function=3). The rules used for target prediction were based on those suggested by Schwab *et al*.^57^,^58^ and Allen *et al*.^57^,^58^. Detailed functional information is important to elucidate the overall expression profiles of potential target genes. Numerous target sequences were assigned to various eggNOG, GO, and KEGG classifications^34^,^35^,^59^.

### RNA extraction and qRT-PCR analysis

Total RNA sequences were extracted using an RNAsimple Total RNA Kit (Tiangen-bio, Beijing, China) according to the manufacturer’s instructions. RNA quality and purity were assessed with OD_260/280_ ratio and determined using a Nanodrop 2000 spectrophotometer (Thermo Scientific, Wilmington, DE). Each sample (10 μg) was reverse transcribed into cDNA using the Prime Script RT reagent Kit (TaKaRa, Dalian, China), and Small RNAs were reverse transcribed into cDNA by using the PrimeScript^TM^ miRNA qPCR Starter Kit Ver.2.0 (TaKaRa, Dalian, China).

qRT-PCR was performed using the ABI 7300 Real-Time PCR System in accordance to the manufacturer’s instructions (SYBR Premix Ex Taq^TM^ and SYBR Green, TaKaRa, Dalian, China). PCR reactions of miRNAs were performed as follows: 95 °C for 30 s, followed by 40 cycles of 95 °C for 5 s, and 60 °C for 30 s. PCR reactions of potential target genes were performed at 94 °C for 30 s, followed by 40 cycles of 94 °C for 10 s and 58 °C for 20 s. A melting curve (61 cycles at 65 °C for 10 s) was generated to check amplification specificity. Each reaction presented three biological repeats, and 5S rRNA and *Agactin* gene were used as internal controls respectively. The primers used for qRT-PCR are listed in Tables 1, 2, 3. *Ct* values were represented by the mean values of three independent replicates, and the relative gene expression levels were calculated using the _△△_Ct method^60^. Standard errors of mean among the replicates were also calculated. Primers were synthesized by GenScript (Nanjing, China).

## Additional Information

**How to cite this article**: Jia, X.-L. *et al*. High-throughput sequencing of small RNAs and anatomical characteristics associated with leaf development in celery. *Sci. Rep*. **5**, 11093; doi: 10.1038/srep11093 (2015).

## Supplementary Material

Supplementary Information

## Figures and Tables

**Table 1:** Primers of five conserved miRNAs used for qRT-PCR analysis.

sense primer	
Agr-miR166	GGAATGTTGTCTGGCTCGAGG
Agr-miR159	GTTTGGATTGAAGGGAGCTCTG
Agr-miR319	TTGGACTGAAGGGAGCTCCC
Agr-miR396	GCTTCCACGGCTTTCTTGAACT
Agr-miR408	TGCACTGCCTCTTCCCTGGC
Agr-miR164	ACGTGCTCCCCTTCTCCAAC

**Table 2:** Primers of five novel potential miRNAs used for qRT-PCR analysis

sense primer	
Agr-miR0056	TGGGTCGGCCTCTACTAACAG
Agr-miR0002	ATGAGAATGGTTGGATCTTT
Agr-miR0005	GGAGCGTCATGCGAACACATC
Agr-miR0046	GTCAGGATGGCCGAGTGGTC
Agr-miR0108	CACGAGCCACTTGGATCATGA

**Table 3:** Primers of corresponding potential target genes used for qRT-PCR analysis.

Gene	Direction	Sequence(5′–3′)	Amplicon size (bp)
*Agr-28401*	Forward	ATGGCTTATCGGCTTCTTTGCTG	112
	Reverse	TGTATCCTTCCTTCTACCCTTGA	
*Agr-20977*	Forward	GGGGTAAGAATGGAGAAGTGTTG	131
	Reverse	TATGCCAGATGAAAGTGTAAGCA	
*Agr-35319*	Forward	CTGTATGAATCACGAGCTTTGCG	83
	Reverse	GTACCATTTGCAGGGACATCAGG	
*Agr-26581*	Forward	AAACTTTCTTCTCAGCCTGGTGC	104
	Reverse	GGACTTGTACTTTCAGATATTAGCG	
*Agr-11109*	Forward	ACAAAGCAACTAAATCACCGACCAC	107
	Reverse	TTACCGTGCCATCGCTCCTGACC	
*Agr-29218*	Forward	CATATCTTCCATCCTCAGGCACG	88
	Reverse	TAGGGCAACTTTAAGGCAGTCCA	
*Agr-55180*	Forward	AAAGACTCGCCTAGTGGAGCAAT	99
	Reverse	GCTCATAAATCCCATAAGACCTG	
*Agr-10763*	Forward	CCCATTCACTGGAACATGCACAG	91
	Reverse	CGACAGCGTTGATACAAATACCG	
*Agr-25317*	Forward	GAGAAACAGGAAGCCAGAAAGCT	99
	Reverse	ACAAGTGCCCCGAACCCTACAAC	
