# Supplementary material for: High-throughput sequencing of small RNAs and anatomical characteristics associated with leaf development in celery
Source: Sci Rep. 2015 Jun 9;5:11093. doi: 10.1038/srep11093 (PMC4460894; doi:10.1038/srep11093)
Supplement: Supplementary Information [file srep11093-s1.pdf]

High-throughput sequencing of small RNAs and anatomical characteristics associated with leaf development in celery

Xiao-Ling Jia, Meng-Yao Li, Qian Jiang, Zhi-Sheng Xu, Feng Wang, Ai-Sheng Xiong*

State Key Laboratory of Crop Genetics and Germplasm Enhancement, College of Horticulture, Nanjing Agricultural University, Nanjing, 210095, China

*Please address all correspondence to: A.S. Xiong (xiongaisheng@njau.edu.cn)

Dr. Ai-Sheng Xiong
Professor
State Key Laboratory of Crop Genetics and Germplasm Enhancement,
College of Horticulture,
Nanjing Agricultural University,
Nanjing, 210095, China
Tel: 86 25 84396790
Fax: 86 25 84396790
Email: xiongaisheng@njau.edu.cn

Supplementary File 1: List of the potential novel miRNAs.

Name	Count	genes	mfe(kcal/mol)
Agr-miR0056	26140	TGGGTCGGCCTCTACTAACAG	-44
Agr-miR0002	1395	ATGAGAATGGTTGGATCTTT	-18.7
Agr-miR0005	754	GGAGCGTCATGCGAACACATC	-35.86
Agr-miR0046	343	GTCAGGATGGCCGAGTGGTC	-28.3
Agr-miR0108	132	CACGAGCCACTTGGATCATGA	-42.75
Agr-miR0030	132	AAGCCAAGGATGATTGCCTGC	-18.76
Agr-miR0105	120	GGACTGTCGGCTGGTTCGAGA	-18.7
Agr-miR0100	68	CGGACTTGGAAGTTGAAACGGT	-24.3
Agr-miR0126	52	AATTTAGTAGGATAAATAGACTAT	-19.4
Agr-miR0089	46	TTTGGCTAATTCAGACGACGTG	-24.82
Agr-miR0001	43	AAGCCAATTTGCAGAGCAAGT	-23
Agr-miR0032	40	CTGAGGCTGATTACATGGCGTG	-22.01
Agr-miR0103	39	TTGCGTGCTCACTGCTCATTCTG	-38.7
Agr-miR0085	36	TCCCTCCCAATTGTTATCGTT	-18.61
Agr-miR0116	35	GGGCATTTGGTCTAGTGGTATG	-18
Agr-miR0021	32	GTTGGAGCTCTCGACGGAGACGGT	-26.7
Agr-miR0090	31	CTCTTCTCGTCATGTTGATCCG	-19.9
Agr-miR0013	30	AACCGGTCGTATTTAACTAAATAC	-34.2
Agr-miR0093	30	CGCATGTGGTGGGGCACCATA	-37.1
Agr-miR0024	29	TGTAACTGTGTAGTATATAAAC	-19.3
Agr-miR0111	24	TCCGATCGGCGCTATCTCGCT	-18.6
Agr-miR0096	22	AATCAGGACTAAGGTGGTTGTC	-18.9
Agr-miR0019	21	ACCGGCTCGTATTCGCTGAATTCG	-22.81
Agr-miR0067	20	AAGGATTTTTCTAGTGTGTGC	-18.5
Agr-miR0003	18	AGAACGAATTTCTGAAAAATCAGG	-31.7
Agr-miR0120	16	GGAGTTGGTCAGAAATTTGGATC	-18.5
Agr-miR0124	16	CGGACGTGTCCAGTCGGTGAATT	-26.9
Agr-miR0039	16	TCTTAGCACTTCCACCTTTGT	-22.5
Agr-miR0081	16	GCTAAGTGGGAAAGGATGTG	-21.5
Agr-miR0016	15	GTGGAGCCTCGTGTGAAGAGC	-24
Agr-miR0051	14	GACTAGCTAGGTCTCTGACCT	-19.4
Agr-miR0117	14	TTTGGATCAGTGAACAGTAGTGAT	-25.7
Agr-miR0031	13	CCGGCTGTAGCTTTGTAGCTC	-21.7
Agr-miR0036	13	AGAGAGGATGAAAGAAGGGAGGCG	-19.4
Agr-miR0064	13	TGTTTGAGGAACAGTCTTTGTC	-19.4
Agr-miR0055	12	ATTGGTTGGAAAATTGAGGTGT	-19.5
Agr-miR0034	12	GAGTTCGGCGAATCAGGACTAA	-23.2
Agr-miR0060	12	TCCTGATGGATTGGATTGGGACGT	-23.14
Agr-miR0118	12	ACGCTTGGGATTGGTCTTACCA	-21.4
Agr-miR0049	11	GGTATACCCGAATCCGATCCGAAA	-34.96
Agr-miR0042	11	TATCCGTCAGCAAGAGCGAATC	-19.4
Agr-miR0059	11	CCGGTCGTTCTAAAAATTTAGGGT	-19.46

Agr-miR0092	11	ATGGTATCAGAGCTTAGGCTG	-23.6
Agr-miR0020	10	GTGTGTGTACGTGTGCCTCTC	-21.7
Agr-miR0018	10	ACTGTCAGTTGGGTATCTCAATT	-19.5
Agr-miR0023	9	ATGGTATCAGAGCTCAGGCC	-21.2
Agr-miR0082	9	ATAAATATGTCGCTGTGTTTTCAG	-29.3
Agr-miR0099	8	AGACGGATCCCCGAGATTCTCCGG	-19.5
Agr-miR0065	7	GTATTGGGTAGAGCAGACTTG	-19.7
Agr-miR0110	7	AGGATGGAAGGACCCTTGCT	-28.4
Agr-miR0083	6	AAATTTGGATTTTTGGATCACAAT	-21.4
Agr-miR0071	6	ATGCACGTAGCACCCCGGGCTTCT	-24.6
Agr-miR0008	6	TGTATCCGATGAATCTCTGTC	-20.2
Agr-miR0062	6	TCAGCTCGACTCAGATGACGGTG	-29
Agr-miR0066	6	TTATTGGTATCTTTGCCTGATC	-18.5
Agr-miR0072	6	GAAGGTCGTTAGAAGTAGAGATGT	-20.5
Agr-miR0080	6	TTGGATTGTTAACATTGTTCA	-25.4
Agr-miR0094	6	AGTGATCTACAGAACTATATGC	-24.6
Agr-miR0104	6	CTATGTTGATCCATCAGATGT	-25
Agr-miR0109	6	TCGCTGAAAAGGCTGAGACTG	-19.1
Agr-miR0102	5	AGAAATGCTGAGCGGCCGCGCAGG	-44.5
Agr-miR0011	5	TGTATCTATGGACATCGTCAT	-20
Agr-miR0076	5	TGAGTGCTATAGCGGACTTGTTAT	-19.4
Agr-miR0091	5	AGGATGGAGATTAAATGGCG	-18.5
Agr-miR0012	5	TTATGGCTCAGATCTACAAA	-25.9
Agr-miR0026	5	TTCATTCATATTAATCGGATT	-30.9
Agr-miR0028	5	GTGAGCCTCTGGGCTGATTAGCTC	-28
Agr-miR0037	5	ATAAATCCTCGAGGGTGTTGCTAC	-20.9
Agr-miR0047	5	ATAAACAAATGGGAGGGACGGAGG	-27.1
Agr-miR0052	5	ATTGAATCTCGAATATGTGTGTGT	-19.1
Agr-miR0061	5	TGTTGTTAAGATCTCATATAG	-30.3
Agr-miR0078	5	GGGCAATACTCTACTGGCAGT	-22.7
Agr-miR0098	5	TGACGTGTTCATTGCTGATGA	-23.1
Agr-miR0101	5	TTCGGCAGGTGAGTTGTTACA	-19.3
Agr-miR0107	5	ATCTAACCTTACATCAGTGTCAAT	-19.5
Agr-miR0114	5	CGGACTTGGACACTTATTTAGGC	-31.22
Agr-miR0130	5	CTACATCTTGGGTATTAGATACTG	-19.3
Agr-miR0131	5	CTTTCCTTGTCAAGTGAGCA	-27
Agr-miR0029	4	TTTTTGGCAAAATACACGGAGG	-20.3
Agr-miR0053	4	CTAGGGTGTGCACATATCAGCTCC	-42.21
Agr-miR0027	4	GAAGGTAAAATGAAGAGTCTGAGT	-18.7
Agr-miR0038	4	AAGTCTAGTGAAAGTTGCTTCATC	-20.6
Agr-miR0050	4	TTTGCGTTCATTGGCACTAGT	-21.9
Agr-miR0069	4	AGGACTGGCTTGGTGATATGACT	-21.3
Agr-miR0077	4	AAAGATGTAGTATCTAACTGGATT	-21
Agr-miR0123	4	ACCGAAGTTGTGGCATTGACA	-20.1

Agr-miR0010	4	TTCTGATGATGATTTCAAATACT	-20.9
Agr-miR0015	4	AAAACTCGGCTCGAGCTCGTTTTG	-25.5
Agr-miR0035	4	ACGGCTTTCTCTGATAGAGGC	-23.7
Agr-miR0040	4	AGCGATCCAATGAGACGATGACGT	-29.5
Agr-miR0048	4	ATTATTCGATGAAACTCCGATCGA	-18.75
Agr-miR0058	4	TGAGGATTTTTTGAAGACGTG	-19.2
Agr-miR0063	4	TTATCTTTCACTTCTAGTAGA	-23.2
Agr-miR0068	4	AGCTATCCTGAGGGAAACTTC	-21.9
Agr-miR0073	4	TAGGCGTGTTGGTGTTTGTCC	-24.7
Agr-miR0074	4	ATCCGACCTTGAACTATCTTG	-22
Agr-miR0075	4	GTGGGCCTTTTGTGCATCTCTACC	-20.6
Agr-miR0084	4	AGTCGATGTCGGTCGATGGTG	-28.2
Agr-miR0128	4	TCGTGGGCTGAAAACGAGATG	-18.8
Agr-miR0009	3	CCTAATGCTAATTATTCAGA	-25.3
Agr-miR0121	3	ATTTCTAGTTTGGGTATTACCATT	-26.8
Agr-miR0007	3	GAAGCGACGGTCCTGAGACTG	-22.8
Agr-miR0054	3	ACTGTTGAAAGCTATGGTTTCGG	-19.7
Agr-miR0057	3	CTTCGCTCTTTATTTGTAATT	-18.5
Agr-miR0088	3	CGGGACCCTCTGGTACCAAGAGTG	-18.5
Agr-miR0095	3	AAGGCTTAGAAGCTTGAGGTG	-23
Agr-miR0097	3	TGGGCTATAGCTCAGTTAGG	-23.8
Agr-miR0122	3	CAGATCTATTCATGTATGTAT	-25.5
Agr-miR0127	3	TTTAGTTTATCTGGTGCTCTAGGC	-20.7
Agr-miR0004	3	AGGGATCTCACTACTCTCAGAAGG	-30.4
Agr-miR0006	3	CAGAGGAGTGGGACTGAGACTG	-20.5
Agr-miR0014	3	GTCTAGGCTCTGACGTTTGTGCTT	-18
Agr-miR0017	3	ATTTATCGGCGATTTTTAAAAAAT	-23
Agr-miR0022	3	AGTGTGTCTTGATAAGAAGCGAGC	-19.6
Agr-miR0025	3	ACATTGTCAAGTGACTCTATCAAG	-24.2
Agr-miR0033	3	TGGACCTGTAGTTGATGCTGC	-26.2
Agr-miR0041	3	TTTGTAACTTATGCCGGAGGT	-19.1
Agr-miR0043	3	ACGATCTACGCCTTGAAGGTATCC	-19.7
Agr-miR0044	3	ATGGTTCAAGGACTAATCGTG	-25.2
Agr-miR0045	3	AACACTGCTCAGATGGATAAGT	-21
Agr-miR0070	3	AGGGTTTAGGATCTAGGATTTAGG	-23.6
Agr-miR0086	3	CGTGTAGGTCCTGGTCAAGACGGA	-23.4
Agr-miR0106	3	TGTCTAAACGGTGTCACTTCTT	-19.5
Agr-miR0112	3	TCCTCGACCGTTCTATTTCTGAGT	-29.6
Agr-miR0113	3	TTTCAGAGTCTTGTAGTTGAG	-20.7
Agr-miR0115	3	TTCAAGATTTCAGGGACACTG	-20.52
Agr-miR0119	3	CTTTAATTGGTCAAGAGCGTG	-23.4
Agr-miR0125	3	AGACTATGCCTTGGTTGGCGC	-19.5
Agr-miR0129	3	TGGTGGATGTTTATACACTGGAGT	-31.74
